# Supplementary material for: Antigen self-anchoring onto bacteriophage T5 capsid-like particles for vaccine design
Source: NPJ Vaccines. 2024 Jan 4;9:6. doi: 10.1038/s41541-023-00798-5 (PMC10766600; doi:10.1038/s41541-023-00798-5)
Supplement: Supplementary file 1 — Supplementary Information [file 41541_2023_798_MOESM1_ESM.pdf]

Antigen self-anchoring onto bacteriophage T5 capsid-like particles for vaccine design

Emeline Vernhes[§1], Linda Larbi Chérif [§2], Nicolas Ducrot[1], Clément Vanbergue[2], Malika Ouldali[1], Lena Zig[2], N'diaye Sidibe[2], Sylviane Hoos[3], Luis Ramirez-Chamorro[1], Madalena Renouard[1], Patrick England[3], Ombeline Rossier[1], Guy Schoehn[4], Pascale Boulanger[1]* & Karim Benihoud[2]*

SUPPLEMENTARY INFORMATION

Supplementary Table 1

Endotoxin and protein content of CLP and pb10-Ova chimera doses administered to mice

	**CLP**			**pb10-Ova chimera**				**Injected dose**		
**Group**	µg/mL	EU/mL	EU/100 µg		µg/mL	EU/mL	EU/100µg	µg/mL	EU/mL	EU/100µg
***pO-CLP**	832	**9.4**	1.1	*pO*	235	**4.6**	2.0	1067	**14.0**	3.1
***pNO-CLP**	832	**9.4**	1.1	*pNO*	200	**8.4**	4.2	1032	**17.8**	5.3
****pNO-CLP**	832	**0.5**	0,06	*pNO*	200	**3.4**	1.7	1032	**3.9**	1.8
*****pNO-CLP**	832	**23.6**	2.8	*pNO*	200	**18.5**	9.3	1032	**42.1**	12.1
*****pCO + CLP**	832	**23.6**	2.8	*pCO*	204	**0.6**	0.3	1036	**24.2**	3.1

EU, Endotoxin Unit
* Samples used to obtain immunization data presented in Figures 3, 4 and 7a
** Samples used to obtain immunization data presented in Figure 5
*** Samples used to obtain immunization data presented in Figures 6 and 7b as well as Supplementary Figure 4

Supplementary Table 2

Oligonucleotides and PCR products used for the construction of expression vectors encoding pb10-mCherry and pb10-Ova chimeras.

PCR product	**Oligonucleotides**		**Vector**
	**Forward Primer*	**Reverse Primer*	
pET28-mC vector	ATGGTGAGCAAGGGCGAG	CATGGTATATCTCCTTCTTAAAGT	
pb10 insert	GAAGGAGATATACCATGGGGATTG	GCCCTTGCTCACCATGCCTCCAGGAACAGTAGG	**pET28-pmC**
pN insert	GAAGGAGATATACCATGGGGATTG	GCCCTTGCTCACCATTGGTGGAGCCGGTGGGG	**pET28-pNmC**
pET28-pb10 vector	CACCACCACCACCACCACTGAG	GCCTCCAGGAACAGTAGGATTAAC	**pET28-pO**
Ova insert	CCTACTGTTCCTGGAGGCATGGGCTCCATCGGC	GGTGGTGGTGGTGGTGAGGGGAAACACATCTGC	
pET28-pN vector	CACCACCACCACCACCACTGAG	TGGTGGAGCCGGTGGGG	**pET28-pNO**
Ova insert	CCACCGGCTCCACCAATGGGCTCCATCGGC	GGTGGTGGTGGTGGTGAGGGGAAACACATCTGC	
pET28-6His vector	AAAA**GGTCTC**A⇣GAATTATCTCCTTCTTAAAGTTAAACAAAATTATTTC	AAAA**GGTCTC**A⇣CACCACCACCACCACCACTGAG	**pET28-pCO**
pCO insert	AAAA**GGTCTC**A⇣ATTCATGTTAACTCTTTCTAAAGATTTAACTGCTAGC	AAAA**GGTCT**CA⇣GGTGAGGGGAAACACATCTGCCAAAG	

In Bold: BsaI recognition sequence. Underlined bases indicate the 4 nucleotides overhangs generated by BsaI cleavage at N1 position (⇣) after the recognition sequence.
*Primers are written in the 5’ to 3’ orientation.

Supplementary Figure 1

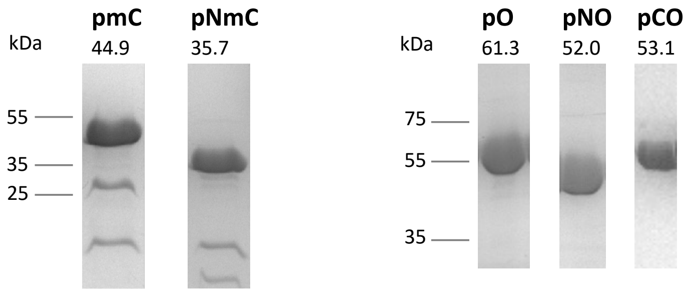

Production of the p10 chimeras. SDS-PAGE analysis of purified pb10-mC and pb10-Ova chimeric proteins eluted from a Superdex 75 10/300 gel filtration column. It should be noted that the bands observed below pmC and pNmC on the gel (left) are not contaminant proteins but results from the slow degradation pmC and pNmC during their storage.

Supplementary Figure 2

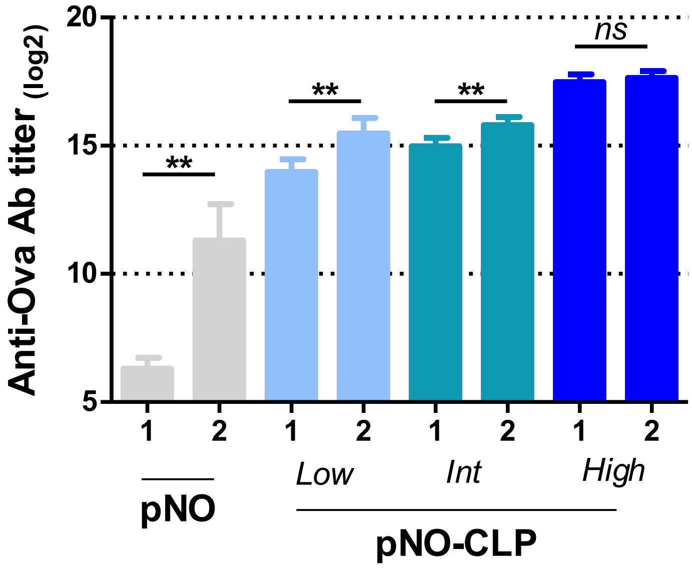

Anti-Ova humoral immune responses elicited by pb10-Ova chimeras after priming and boosting. C57BL/6 mice were immunized twice subcutaneously with pNO alone or with different concentrations of pNO-CLP (low, intermediate and high dose). Titers of Ova-specific IgG were determined by ELISA at day 28 after the first (1) and the second (2) administration. Mean + SEM, n = 6. Results were analyzed using paired t-test with $p < 0.05$ considered significant (*ns*, non-significant; **, $p < 0.01$).

Supplementary Figure 3

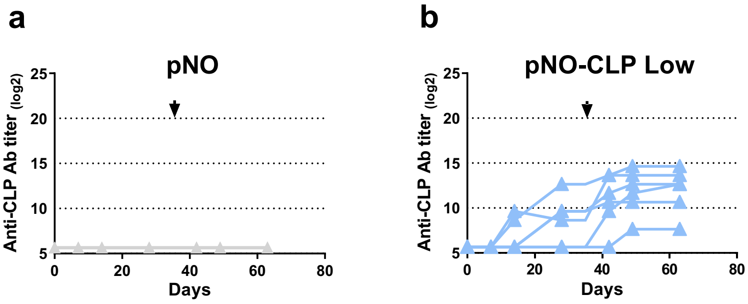

Experimental group	pNO (µg)	CLP (µg)
pNO-CLP High	10.0	41.0
pNO-CLP Int	1.0	4.1
pNO-CLP Low	0.1	0.4
pNO	10.0	-

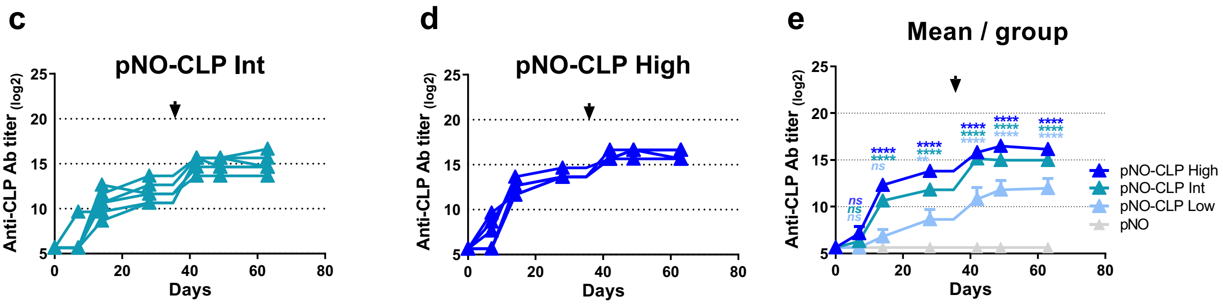

Dose-dependent anti-capsid humoral immune responses elicited by pb10-Ova chimeras. C57BL/6 mice were immunized twice subcutaneously with pNO alone or with different concentrations of CLP-pNO (low, intermediate and high dose). Day 35 corresponds to the second administration (arrow). Titers of CLP-specific IgG were determined by ELISA at different time points after injection. Titers below 100 were plotted as $\log_2(50)$. (**a-d**) Each curve represents titers ($\log_2$ scale) of individual mice (n = 6) of the indicated group. (**e**) Comparison of the kinetics of the different groups (mean + SEM). Two-way repeated measures ANOVA was used for comparison of responses to pNO alone, at different time points, then Bonferroni post hoc test was used with $p < 0.05$ considered significant (*ns*, non-significant; *, $p < 0.05$; **, $p < 0.01$; ***, $p < 0.001$; ****, $p < 0.0001$).

Supplementary Figure 4

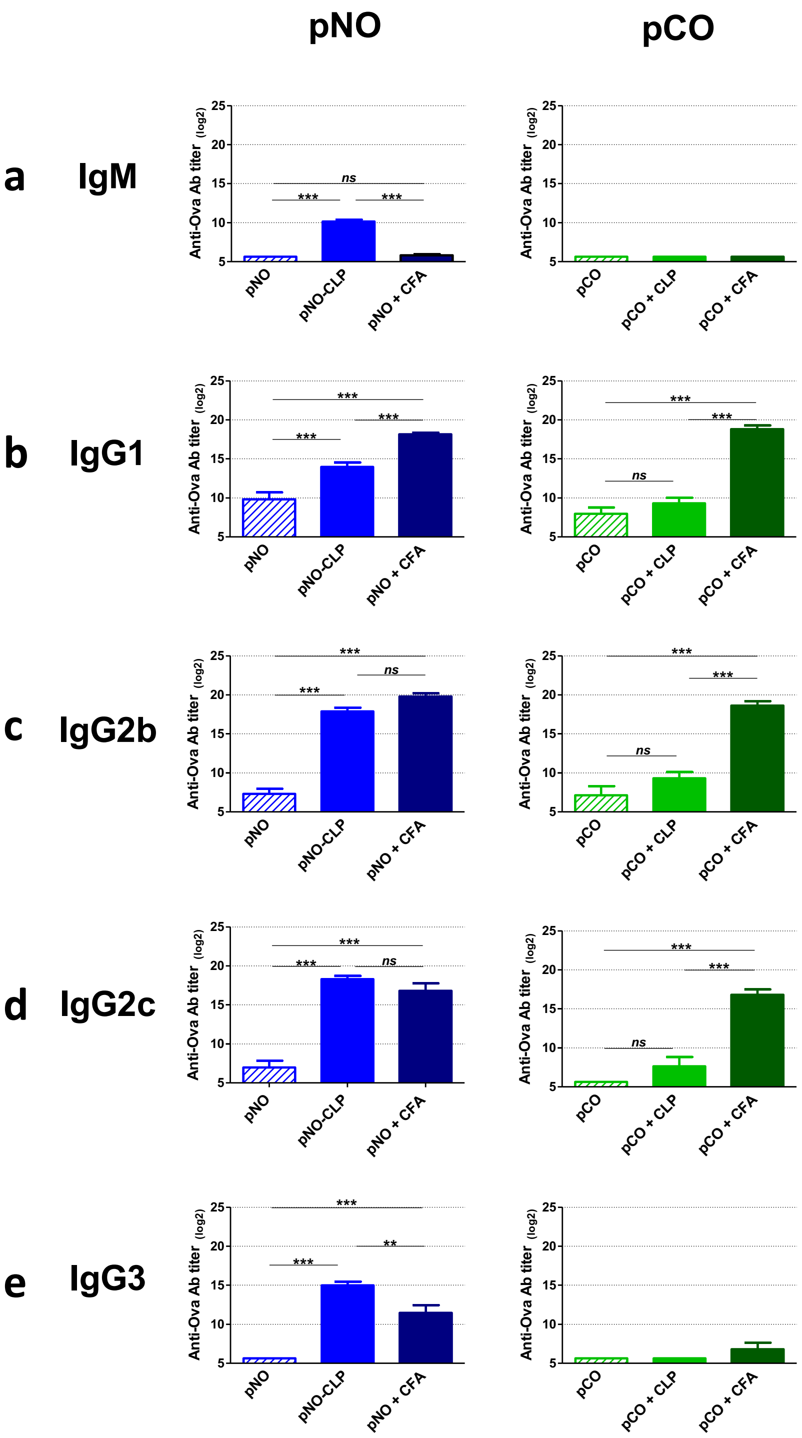

Nature of anti-Ova antibodies elicited by pNO and pCO chimeras. Mice were immunized subcutaneously with pb10 chimeric proteins (**Left**, pNO; **Right**, pCO) alone or in combination with either T5 CLPs or CFA. Titers of anti-Ova IgM (**a**), IgG1 (**b**), IgG2b (**c**), IgG2c (**d**) and IgG3 (**e**) Ab isotypes were determined by ELISA at day 42 p.i. The results correspond to the mean + SEM of each group (n = 6, log_2 scale). Titers below 100 were plotted as $log_2(50)$. Results were analyzed using one-way ANOVA followed by Tukey's multiple comparison test with $p < 0.05$ considered significant (*ns*, non-significant; ****, $p < 0.01$; *****, $p < 0.001$).

Supplementary Figure 5

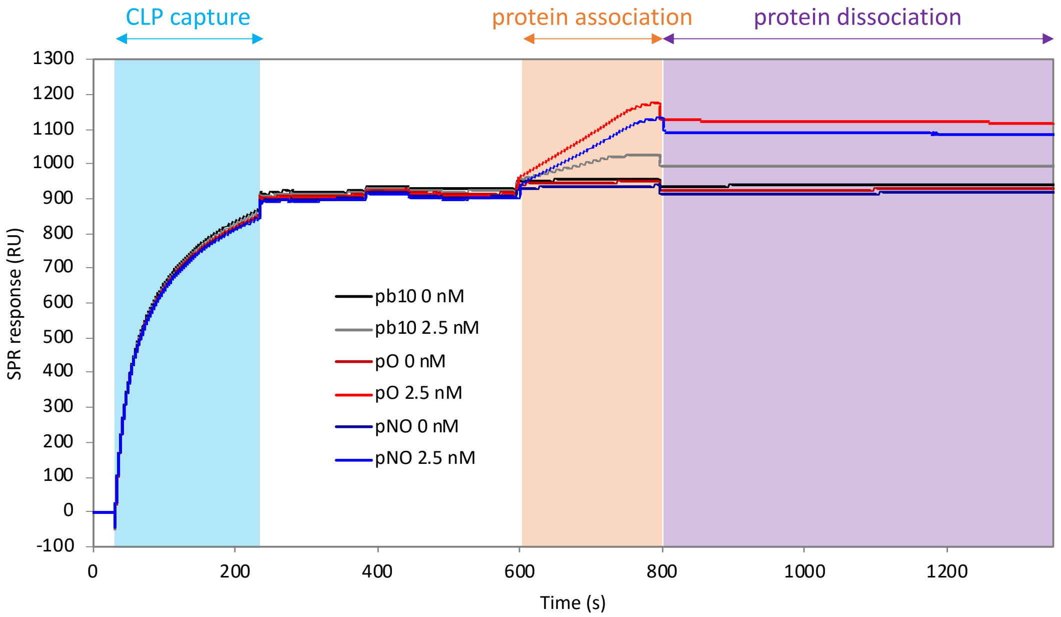

Typical SRP profiles of CLP capture (200 s) followed by association (200 s) and dissociation (550 s) of pb10 chimeras. Real-time SPR sensorgrams (resonance units, RU) obtained with 0 nM (control with the protein-free buffer) or 2.5 nM (saturating concentration) of pb10, pO and pNO. Analyzed data presented in **Figure 1d** include subtraction by the 0 nM curves. Note that pb10, pO and pNO associations induce different levels of response due to the proteins' different molecular weights. These differences were considered for the calculation of occupied binding sites in **Figure 1**, as described in the Methods.
