# Supplementary figures and images for: Antigen self-anchoring onto bacteriophage T5 capsid-like particles for vaccine design
Source: NPJ Vaccines. 2024 Jan 4;9:6. doi: 10.1038/s41541-023-00798-5 (PMC10766600; doi:10.1038/s41541-023-00798-5)

Figure 1-e

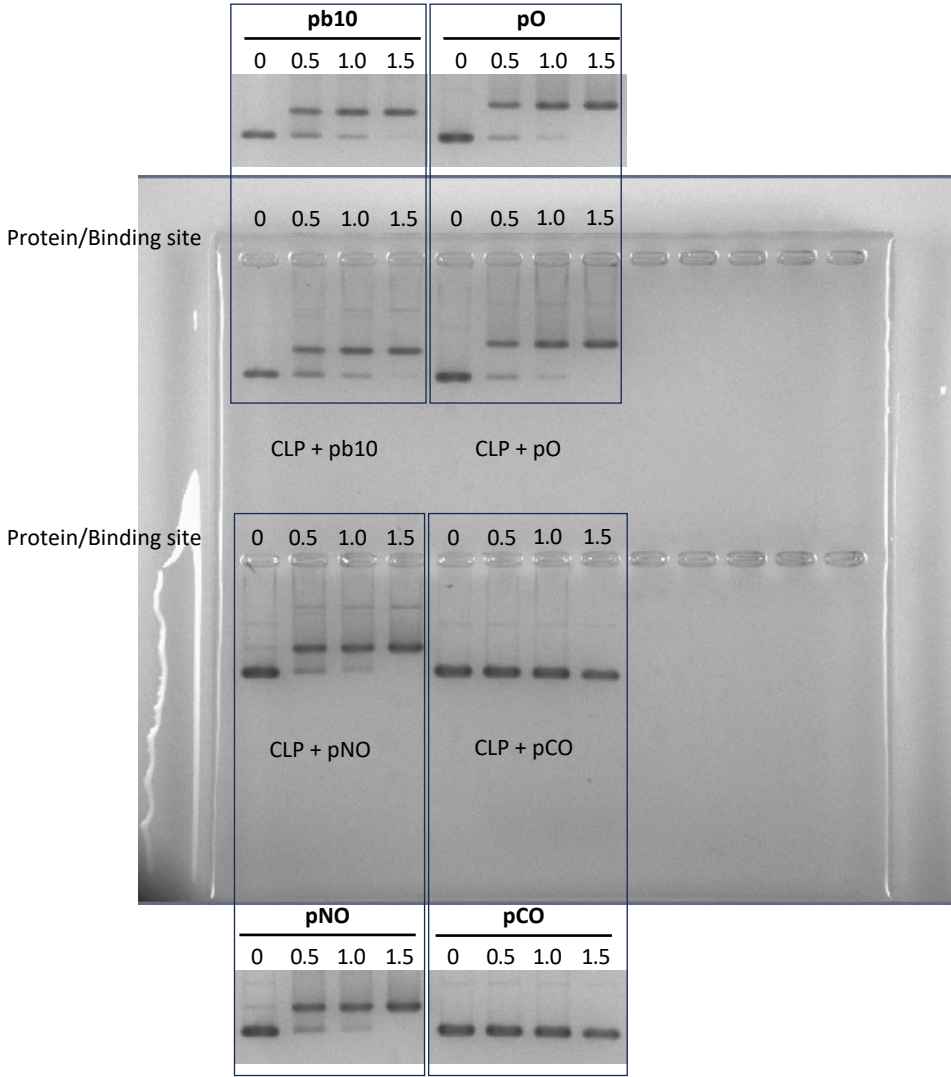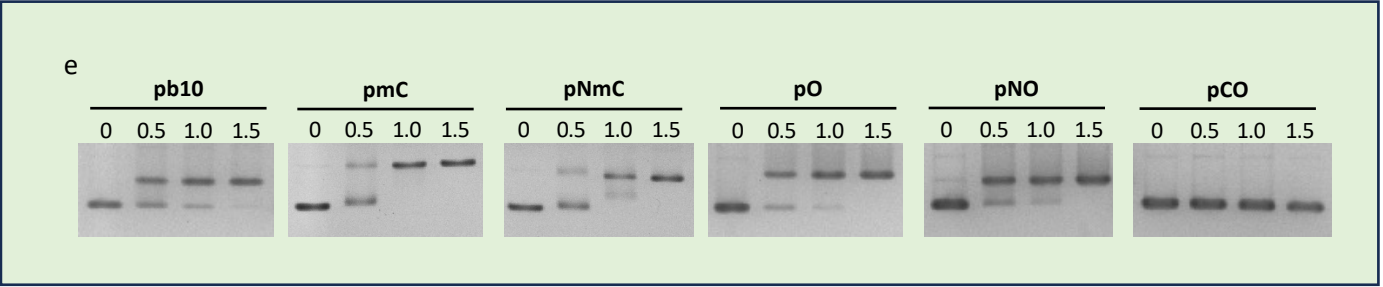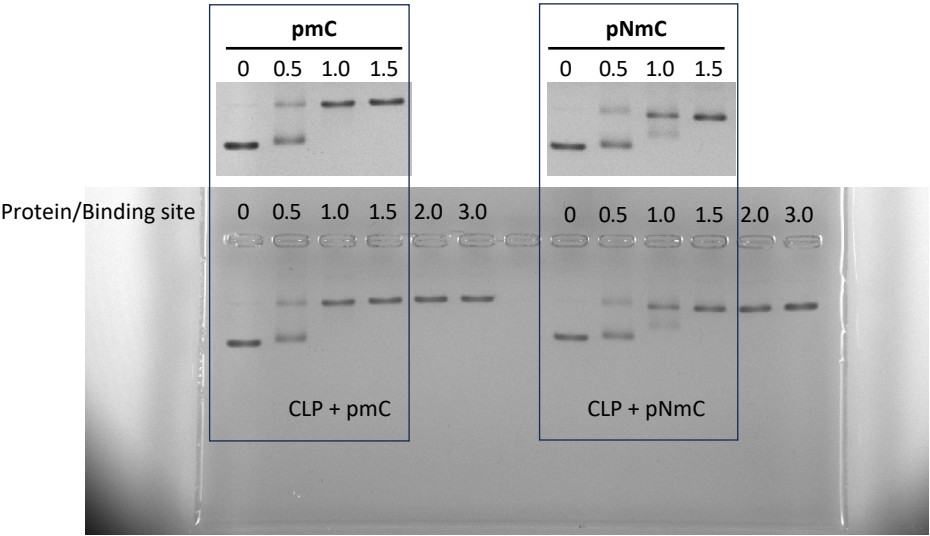

Supplement: Supplementary file 2 — DATAset 1 [file 41541_2023_798_MOESM2_ESM.pdf]
