# Supplementary material for: Antigen self-anchoring onto bacteriophage T5 capsid-like particles for vaccine design
Source: NPJ Vaccines. 2024 Jan 4;9:6. doi: 10.1038/s41541-023-00798-5 (PMC10766600; doi:10.1038/s41541-023-00798-5)
Supplement: Supplementary file 3 — DATAset 2 [file 41541_2023_798_MOESM3_ESM.pdf]

NPJVACCINES-02584R

Figure S1-b

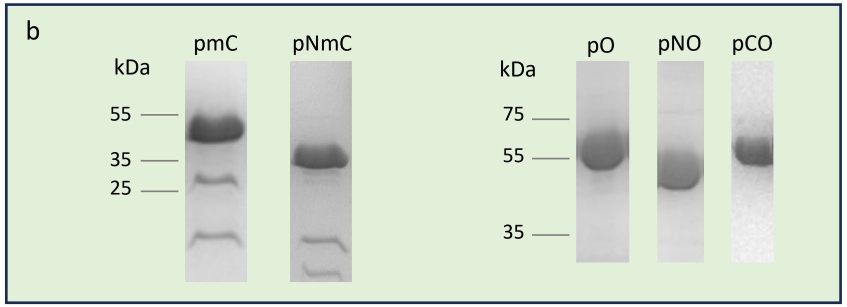

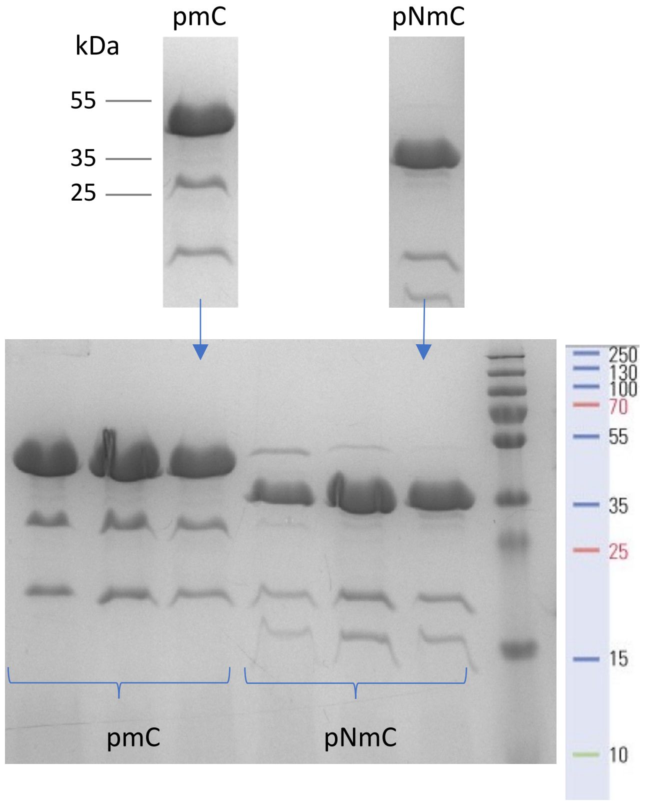

Fractions of pmC and pNmC
Superdex 75 purification

Thermo Scientific™ PageRuler™
Plus Prestained Protein Ladder,
10 to 250 kDa

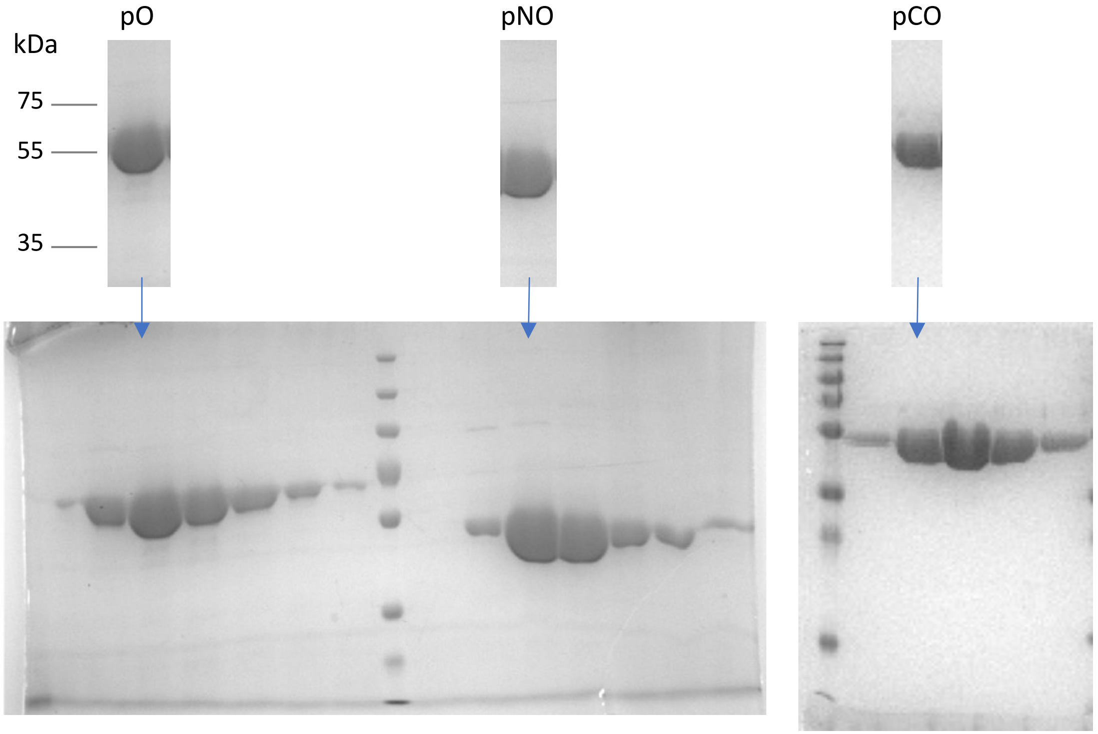

Fractions of pO, pNO and pcO
Superdex 75 purification
